# Reduced synaptic tagging by complement protein C3 is associated with elevated extracellular matrix in the middle-aged cerebellum of mice

**DOI:** 10.3389/fnagi.2025.1616390

**Published:** 2025-06-26

**Authors:** Henning Peter Düsedau, Carla Cangalaya, Stoyan Stoyanov, Alexander Dityatev, Ildiko Rita Dunay

**Affiliations:** ^1^Institute of Inflammation and Neurodegeneration, Health Campus Immunology, Infectiology and Inflammation (GC-I^3^), Otto-von-Guericke-University, Magdeburg, Germany; ^2^Molecular Neuroplasticity Research Group, German Center for Neurodegenerative Diseases (DZNE), Magdeburg, Germany; ^3^Center for Behavioral Brain Sciences (CBBS), Magdeburg, Germany; ^4^Medical Faculty, Otto-von-Guericke-University, Magdeburg, Germany; ^5^German Center for Mental Health (DZPG), Magdeburg, Germany; ^6^Center for Intervention and Research on Adaptive and Maladaptive Brain Circuits Underlying Mental Health (C-I-R-C), Halle-Jena-Magdeburg, Germany

**Keywords:** extracellular matrix, proteoglycans, aging, microglia, cerebellum, complement system, synaptic pruning, synaptosomes

## Abstract

**Background:**

Aging of the brain is associated with cognitive decline and recognized as a major risk factor for the development of neurodegenerative diseases. On a cellular level, brain aging is accompanied by a progressive increase of the basal pro-inflammatory tonus, leading to the activation of phagocytic pathways in brain-resident microglia and disruptive effects on synaptic neurotransmission. While the aging process affects all brain compartments at different velocities and one of the particularly affected regions is the cerebellum (CB), the underlying effects remain elusive.

**Methods:**

In the present study, we harnessed a murine model of natural aging in males combined with orthogonal experimental approaches comprising of cytokine gene expression analysis, flow cytometry, immunohistochemistry, and flow synaptometry.

**Results:**

We report age-dependent morphological and phenotypic changes in microglia that are distinct in the cortex (CTX) and CB. Furthermore, we show an increased expression of cytokines and complement factors upon aging and a decline of C3-tagged VGLUT1^+^ presynaptic puncta in the CB. Using flow synaptometry to quantify the composition of synapses in more detail, we validated the reduction of C3b-labeled excitatory synaptosomes while the overall frequency of glutamatergic synaptosomes remained unaffected by aging. Notably, proteoglycans brevican and aggrecan, key components of the neural extracellular matrix, were significantly upregulated in the middle-aged CB.

**Discussion:**

The data presented herein suggests the ECM-mediated shielding of synapses from complement-tagging and subsequent engulfment by microglia. Thus, we provide novel insights into mechanisms that may confer resilience in the brain by modulating synapse removal in the context of aging.

## 1 Introduction

Aging is an inescapable biological process that leads to a gradual decline in physiological and cognitive functions. Although advancements in healthcare and living conditions have significantly increased life expectancy in modern societies, population aging has become a prominent issue that poses significant challenges, including the impact on healthcare resources and quality of life. In the central nervous system (CNS), normal aging is marked by structural changes due to the constant reduction of grey matter (Aljondi et al., 2019) but also cellular alterations that impact gene expression, metabolism, and synaptic plasticity (Bettio et al., 2017). Consequently, aging remains to be recognized as a major risk factor for the development of neurodegenerative diseases (Adamczyk-Sowa et al., 2022; Costantini et al., 2018; Jurcau et al., 2024; Ajoolabady et al., 2024). One region of the brain particularly affected by the aging process is the cerebellum (CB), which plays a crucial role in motor coordination, balance, and cognitive processing. This decline in cerebellar function has been associated with the onset of motor deficits, such as decreased coordination and gait disturbances, as well as cognitive impairments and memory (McElroy et al., 2024). Noteworthy, the CB has been shown to display disproportional loss of Purkinje neurons and age-related impairment in long-term depression in mice (Woodruff-Pak et al., 2010), but the underlying effects have not been fully explored.

In the healthy brain, microglia represent the predominant resident population of immune cells and perform a variety of tasks. Given their origin as yolk sac-derived macrophages (Ginhoux et al., 2010), microglia are capable of removing cellular debris and modulating the complex network of proteins and carbohydrates that form the extracellular matrix (Nguyen et al., 2020). Moreover, microglia take a central role in sculpting the brain’s neuronal network by engulfing and removing weak or surplus synaptic inputs with the involvement of the complement system (Stevens et al., 2007; Schafer et al., 2012; Neniskyte and Gross, 2017). Upon infection or tissue damage, microglia act as the first line of defense and respond by the release of inflammatory mediators, such as interleukin (IL)-1β, IL-6, complement factors, and tumor necrosis factor (TNF) in addition to chemoattractants that stimulate the recruitment of peripheral immune cells, ultimately culminating in neuroinflammation (Colonna and Butovsky, 2017; Wolf et al., 2017). However, if not self-limited, chronic and persistent neuroinflammation is considered detrimental and associated with several neuropsychiatric and neurodegenerative diseases (Zhang et al., 2023; Heneka et al., 2015). This is further supported by the observation of a re-activated excessive pruning of viable synapses by aberrantly and irregularly activated microglia during neuroinflammatory conditions (Hong et al., 2016; Vasek et al., 2016). Notably, brain aging is accompanied by chronic, low-grade neuroinflammation driven by a progressive increase in the inflammatory tone of the innate immune system (Franceschi et al., 2018). Aging is, therefore, linked to the emergence of cellular senescence and age-dependent dysregulation of microglia (Melo Dos Santos et al., 2024; Damani et al., 2011; Sikora et al., 2021).

Despite the remarkable progress that has been made in the understanding of the transcriptomic and proteomic landscape of the brain, the susceptibility of the CB to age-dependent cognitive decline is still not fully understood. Here, we demonstrate in a mouse model of natural aging that microglia undergo distinct morphological and phenotypic changes in subdomains of the cortex (CTX) and CB. Utilizing a multifaceted research approach encompassing immunohistochemistry and flow cytometry, our study underscores the increased expression of lysosomal and phagocytosis-associated markers by microglia in the middle-aged CB, concomitant with elevated transcript levels of complement factors *C1qa* and *C3*. Multi-parametric flow synaptometry, however, showed a reduced number of C3-tagged VGLUT1^+^ synaptic terminals in the middle-aged CB. In line with these findings, no changes were observed in the overall abundance of glutamatergic synapses, ruling out the active removal of excitatory synapses by activated microglia. Lastly, our study highlights the marked increase of aggrecan and brevican expression in the CB of naturally aged mice, proposing an ECM-dependent mechanism to interfere with complement-dependent synapse removal in the process of aging.

## 2 Materials and methods

### 2.1 Animals

In this study, experiments were performed with young (12-weeks-old) and naturally aged (1-year-old) male C57BL/6 mice, originally purchased from Jackson Laboratory (Bar Harbor, ME, United States) and all bred in the animal facility of the DZNE site in Magdeburg, Germany. Animals were group-housed for the respective time period under specific pathogen-free conditions in individual-ventilated cages with a 12 h day/night cycle with free access to food and water. All animal care was in accordance with institutional guidelines and experiments were performed in accordance with the German National Guidelines for the Use of Experimental Animals and the protocol approved by the Landesverwaltungsamt Saxony-Anhalt (42502-2-1346 DZNE).

### 2.2 Cell isolation

Tissue collection and cell isolation were performed as described previously (Düsedau et al., 2021). In brief, mice were deeply anesthetized with Isoflurane (CP Pharma, Burgdorf, Germany) and perfused intracardially with 60 mL sterile phosphate-buffered saline (PBS) prior to brain extraction. For subsequent analysis by flow cytometry, brains were dissected into cortex (CTX) and cerebellum (CB) according to the Allen Mouse Brain Atlas (Lein et al., 2007), collected in separate tubes and homogenized in a buffer containing Hank’s balanced salt solution (HBSS, Gibco, New York, NY, United States), 13 mM HEPES (pH 7.3, Thermo Fisher Scientific, Waltham, MA, United States) and 0.68% glucose before sieving through a 70 μm cell strainer. The homogenate was fractioned on a discontinuous isotonic 30–70% Percoll gradient (GE Healthcare, Chicago, IL, United States). Immune cells were collected from the 30/70% Percoll interphase, washed in PBS and immediately processed for subsequent flow cytometric analysis.

### 2.3 RNA isolation

Tissue samples were collected as described above and stored in RNAlater solution (Sigma-Aldrich, #R0901). Isolated brain regions were homogenized in lysis buffer using BashingBeads Lysis tubes (Zymo Research Europe, Freiburg, Germany) and RNA was isolated using AllPrep DNA/RNA Mini Kit (Qiagen, Hilden, Germany) according to the manufacturer’s instructions. Concentration and purity of isolated RNA was determined using a NanoDrop 2000 spectrophotometer (Thermo Fisher) and RNA was stored at -80°C until further use.

### 2.4 Reverse transcription qPCR

Gene expression levels were assessed using 30 ng isolated RNA. Amplification was carried out with TaqMan^®^ RNA-to-CT™ 1-Step Kit (Applied Biosystems, Foster City, CA, United States) and LightCycler^®^ 96 (Roche, Basel, Switzerland) as previously described (Figueiredo et al., 2022). Thermal-cycling parameters were set as follows: reverse transcription (48°C, 30 min), enzyme activation (95°C, 10 min) followed by 55 cycles of denaturation (95°C, 15 s) and annealing/extension (60°C, 1 min). Utilized TaqMan^®^ Gene Expression Assays (Applied Biosystems) are listed in Table 1. For relative quantification, expression of *Hprt* was chosen as reference and relative target gene mRNA levels were determined by the ratio target gene / reference gene and subsequently normalized to the mean values of young cortex group.

**Table 1:** TaqMan^®^ assays used for RT-qPCR analyses.

Protein	Gene	Assay ID
C1qa	*C1qa*	Mm00432142_m1
C3	*C3*	Mm01232779_m1
HPRT	*Hprt*	Mm01545399_m1
IL-1β	*Il1b*	Mm00434228_m1
IL-6	*Il6*	Mm00446190_m1
TGF-β	*Tgfb1*	Mm01178820_m1
TNF	*Tnf*	Mm00443258_m1
TREM2	*Trem2*	Mm04209422_m1

### 2.5 Immunohistochemistry

Mice were deeply anesthetized and perfused as described above, then isolated brains were dissected and fixed in 4% paraformaldehyde-phosphate buffer. Subsequently, brains were cryoprotected in 30% sucrose and stored in 100% 2-methylbutane at -80°C. 50 μm-thick coronal sections were prepared using a microtome and stored at 4°C, floating in a solution containing one part ethylene glycol, one part glycerin, and two parts PBS (pH = 7.2). Coordinates to identify different brain regions were determined using the Franklin and Paxinos (2008) mouse brain atlas in sagittal sections (Franklin and Paxinos, 2008). The motor cortex (MC) was located between ML 2.04, AP 2.8 to 1 mm, DV 0.5 to 2 mm, while the somatosensory cortex (SC) was located between ML 2.04, AP 1.5 to -1.5 mm, DV 0.0 to 1.2 mm. The retrosplenial cortex (RSC) was found around ML 0.48 mm, AP -1.0 to -4.0 mm, and DV 0 to 1 mm. The deep cerebellar nuclei (DCN) were located around coordinates ML 2.04, AP -6.0 mm, DV 3 mm, and the cerebellar cortex (CC) above the DCN was located around ML 2.04, AP -5.0 to -8.0 mm, DV 1 to 3 mm. For immunostaining, sections were first washed in PBS three times for 10 min each, followed by blocking in 5% normal goat serum (NGS) (Gibco, Cat# 16210-064) with 0.5% Triton X-100/PBS for 1 h at room temperature. Sections were then incubated for 48 h at 4°C with primary antibodies diluted in the blocking solution: anti-IBA1 (1:500, rabbit, Cat# 019-19741, Wako), anti-CD68 (1:500, rat, Cat# MCA1957, Bio-Rad), anti-VGLUT1 (1:1000, chicken, Cat# 135316, Synaptic Systems), anti-Homer1 (1:500, guinea pig, Cat# 160004, Synaptic Systems), anti-brevican (1:500, guinea pig) (John et al., 2006), anti-C3 (1:500, rat, Cat# HM1045, Hycultec), and anti-aggrecan (1:000, rabbit, Cat#AB1031, Merck Millipore). After primary antibody incubation, sections were washed again in PBS three times for 10 min each. Sections were then incubated with secondary antibodies (goat anti-rabbit Alexa Fluor™ 488, 1:500; goat anti-rat Alexa Fluor™ 647, 1:500; goat anti-chicken Alexa Fluor™ 547, 1:500; Invitrogen) for 3 h at room temperature on a shaker. Following a final wash in PBS three times for 10 min each, sections were mounted on Superfrost Plus slides with Fluoromount mounting media (Sigma-Aldrich, Cat# F4680). Confocal microscopy was performed with a Zeiss LSM 700 microscope equipped with EC Plan-Neofluar 20x objective (1024×1024 pixels, 10 optical sections, 1 μm intervals between sections).

### 2.6 Histochemical measurements

For the quantification of microglial IBA1 area (%), three 20x images of different cortical and cerebellar areas were acquired per mouse. Z-stacks were converted to maximum intensity projections and IBA1 staining was automatically thresholded (Otsu dark) using FIJI software. Particles were analyzed to determine IBA1^+^ area (Baligács et al., 2024).

CD68, brevican, and aggrecan intensity were measured in brain regions of interest using confocal microscopy at a 20x objective, with comparable sections from each animal. Mean intensity for all antibodies (CD68, brevican, and aggrecan) was quantified using FIJI. For microglial lysosome size quantification, 4–6 z-stacks were acquired at a 20x objective, maintaining consistent microscope settings across all samples. Z-stacks were then converted to maximum intensity projections using FIJI/ImageJ. An intensity threshold for CD68 quantification was manually selected and applied to all sections. Following thresholding, particles were analyzed, and relative area, number, and average size were recorded.

For quantification of VGLUT1^+^ HOMER1^+^ and C3-labeled synapses, 4–6 z-stacks were obtained with a 63x (NA 1.4) oil immersion objective. Microscope settings were kept the same for all samples that were compared. The numbers of VGLUT1^+^ HOMER1^+^ puncta and synaptic C3-puncta were automatically quantified using a FIJI Synapse Counter plugin (Dzyubenko et al., 2016), the latter was defined by counting C3^+^ puncta colocalized with VGLUT1^+^ puncta.

### 2.7 Flow cytometric analysis

For flow cytometric analysis of cell phenotypes, freshly isolated cells were first incubated with Zombie NIR™ fixable dye (BioLegend, CA, United States) for live/dead discrimination and with anti-FcγIII/II receptor antibody (clone 93) to prevent unspecific binding of antibodies. Cells were further stained with the following fluorochrome-conjugated antibodies against cell surface markers in FACS buffer (PBS containing 2% fetal bovine serum and 0.1% sodium azide): eFluor™ 450-F4/80 (clone BM8) and FITC-MHC Class I (clone 28-14-8), APC-CD11b (clone M1/70) (purchased from eBioscience™, San Diego, CA, United States), Brilliant Violet™ 510-CD45 (clone 30-F11), PerCP/Cy5.5-CD80 (clone 16-10A1), PE/Dazzle594™-CX3CR1 (clone SA011F11) (purchased from BioLegend), and PE-TREM2 (clone 237,920) (purchased from R&D Systems Inc., Minneapolis, MN, United States). Cells were acquired using an Attune NxT flow cytometer (Thermo Fisher) and obtained data were analyzed using FlowJo software (version 10.5.3, FlowJo LLC, OR, United States). Fluorescence Minus One (FMO) controls were used to determine the level of autofluorescence.

### 2.8 Synaptosome isolation and flow synaptometry

Tissue samples from CTX and CB were harvested as described above, snap-frozen in liquid nitrogen and stored at -80°C. Isolation of crude synaptosomes was performed as described elsewhere (Düsedau et al., 2021) and freshly isolated synaptosomes were resuspended in SET buffer (320 mM sucrose, 1 mM EDTA, 5 mM Tris, pH 7.4) with 5% DMSO, aliquoted, slowly frozen (-1°C / min), and stored (-80°C) until further use (Hobson and Sims, 2019). Thawed synaptosomes were centrifuged (14,000 *g*, 10 min, 4°C), fixed and permeabilized (Foxp3 / Transcription Factor Staining Buffer Set, 45 min, 4°C), washed with SET buffer, centrifuged (14,000 g, 10 min, 4°C), and resuspended in permeabilization buffer with 10% normal goat serum (heat-inactivated). Primary antibodies against synaptophysin (1:2000, Cat# 101308, Synaptic Systems), synaptobrevin 2 (1:1000, Cat# 104318, Synaptic Systems), GluR1 (1:400, Cat# 13185, Cell Signaling Technology), GluR1 (1:400, Cat# ABN241, Merck Millipore), and C3b (1:200, Cat# MA1-70053, Thermo Fisher) were added and incubated (45 min, 4°C), washed, and co-stained (45 min, 4°C) with: goat anti-guinea pig AlexaFluor™ 546 (1:200, Cat# A11074, Thermo Fisher), goat anti-rabbit AlexaFluor™ 488 (1:4000, #A32731, Thermo Fisher), goat anti-mouse AlexaFluor™ 405 (1:200, Cat# A31553, Thermo Fisher). Synaptosomes were washed, stained with FM™4-64FX (0.2 μg/mL, Cat# F34653, Thermo Fisher), diluted, and acquired using an Attune™ NxT flow cytometer. Data were analyzed using FlowJo.

### 2.9 Statistical analysis

Statistical analysis of data was done using GraphPad Prism 7 (GraphPad Software, CA, United States). Results of flow cytometry were z-score normalized and subsequently plotted as a heatmap using R (version 4.5.0) (R Core Team, 2025) with “lattice” package (Sarkar, 2008). Data shown are representative of three independent experiments. In all cases, results are presented as arithmetic mean and were considered significant, with *p* < 0.05.

## 3 Results

**Figure 1:**
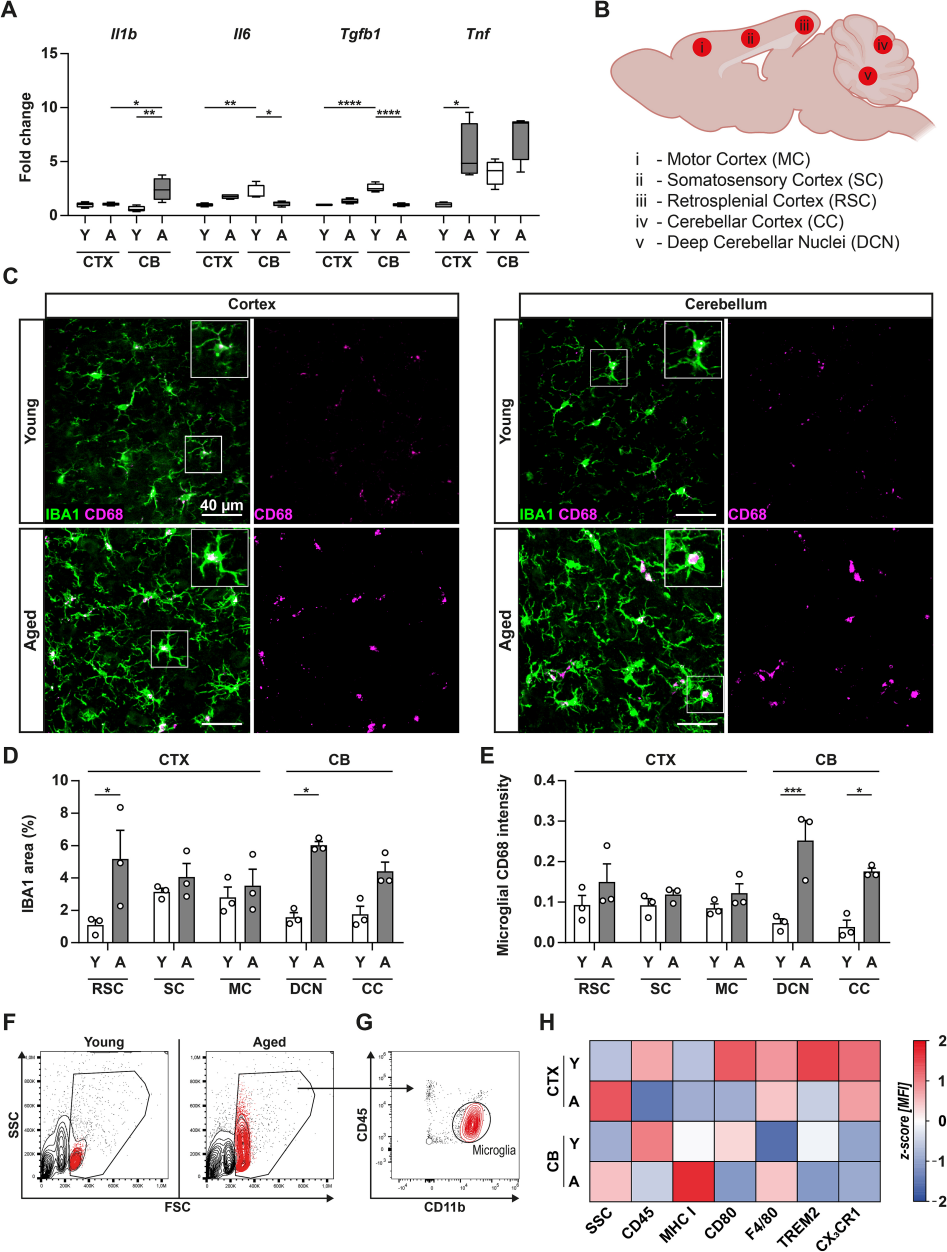
Aging induces region-specific upregulation of phagocytosis markers in microglial cells. **(A)** Gene expression levels of IL-1β (*Il1b*), IL-6 (*Il6*), TGF-β (*Tgfb1*), and TNF (*Tnf*) in cortex (CTX) and cerebellum (CB) of young (white bars) and middle-aged (grey bars) mice were examined using RT-qPCR. Expression levels of target genes were normalized to the expression level of *Hprt* and subsequently, relative expression was normalized to the mean expression level in CTX of young animals to obtain the fold change. **(B)** Overview of subregions in CTX and CB used for immunohistochemistry of microglial cells. **(C)** Panels depict immunofluorescence imaging of microglial cells stained with antibodies against IBA1 and CD68 in CTX (left) and CB (right) of young (upper panels) or middle-aged (bottom panels) mice. **(D,E)** Bar charts illustrate quantified data from **(C)** for the different subregions of CTX and CB. **(F–H)** Flow cytometric characterization of microglia isolated from CTX and CB of young and middle-aged mice. **(F,G)** Gating strategy applied for the identification of microglia. First, cells were selected based on forward scatter/side-scatter light properties (FSC/SSC), displaying distinct alterations between young and middle-aged cells. **(G)** Upon exclusion of dead cells, only CD45^+^ events were selected (not shown) and subsequently gated for CD45/CD11b expression. Microglia were selected as CD45^+^CD11b^+^ cells, representing the largest population of immune cells in both CTX and CB. **(H)** Heatmap of z-score-normalized microglial surface marker expression and SSC properties. Data are shown as mean ± SEM and were compared by two-way ANOVA with Tukey’s *post-hoc* correction. Significant differences are indicated by * *p* < 0.05, ** *p* < 0.01, *** *p* < 0.001, **** *p* < 0.0001.

Previously, aging of the brain has been shown to be accompanied by progressive elevation of innate cytokine expression levels (Franceschi et al., 2018). To study the susceptibility of the CB to aging, we used a murine model of natural aging and compared the gene expression of IL-1β, IL-6, TNF, and TGF-β in the CTX and CB of young (12-weeks-old) and naturally middle-aged (1-year-old) male C57BL/6 mice (Figure 1A). We detected that aging induced a prominent increase in *Tnf* expression levels in the CTX that was accompanied by a similar, although not significant, trend in the CB (*p* < 0.085). We also observed an upregulation of *Il1b* expression, but only in the CB of middle-aged animals, while levels of *Il1b* expression in middle-aged CTX samples remained unaltered. Interestingly, gene expression analysis revealed a striking difference in the abundance of both *Il6* and *Tgfb1* mRNA copies in young animals with elevated levels found in the CB compared to CTX, indicating a divergent development upon aging. Whereas *Il6* expression levels in the CTX tended to increase with age (*p* < 0.0757), levels in the CB were significantly decreased upon aging. Similarly, *Tgfb1* expression was considerably reduced between young and middle-aged CB. Taken together, RT-qPCR analysis of cytokine gene expression levels indicated notable differences in young and middle-aged CTX and CB.

As these cytokines are attributed to the innate immune system (Lacy and Stow, 2011), we wondered whether aging asymmetrically affected the activation of microglial cells in these two regions. Consequently, microglia were examined on a cellular level by immunofluorescence microscopy (IF) in distinct regions of the CTX (motor cortex, somatosensory cortex, and retrosplenial cortex) and CB (cerebellar cortex and deep cerebellar nuclei) (Figure 1B). We utilized the pan-myeloid marker Ionized calcium-binding adaptor molecule 1 (IBA1) to label microglia and detected that aging induced an upregulation in IBA1 levels in the retrosplenial cortex (RSC) and deep cerebellar nuclei (DCN) (Figures 1C–E). In addition, the signal overlap between IBA1 and the lysosomal marker CD68 was significantly elevated in two studied CB subregions, DCN and cerebellar cortex (CC), consistent with an increased phagocytic capacity of microglia. However, no change was observable in the studied CTX subregions. Since microglia activation states are heterogeneous, complex, and dynamic (Paolicelli et al., 2022), we next performed multi-parametric flow cytometry of freshly isolated brain cells. We identified microglial cells based on the expression levels of CD45 and CD11b as CD45^int^CD11b^+^ (Figures 1F,G) and observed regional differences in the frequency of these cells in CTX and CB (Supplementary material A), in addition to higher internal complexity of these cells upon aging, i.e., greater values measured in the side-scatter light (SSC) parameter (Figure 1H). Nonetheless, microglia represented the major population of immunocytes in all experimental groups and we consequently sought to characterize their expression levels of surface receptors associated with function and activity levels (Figure 1H; Supplementary material B–G). We detected regional and aging-dependent differences in the microglial surface expression of CD45, CD80, TREM2, and CX3CR1. Interestingly, cerebellar microglia displayed a contrasting pattern to cortical microglia by showing a substantial increase in major histocompatibility I (MHC I) and F4/80 expression levels upon aging, thereby reconfirming our microscopy results and a greater phagocytic activity when compared to cells isolated from middle-aged CTX. TREM2 plays a role in neurodegenerative diseases where its elevated expression is a hallmark of disease-associated microglia (Keren-Shaul et al., 2017; Deczkowska et al., 2018), but it can also be cleaved from cell surfaces. With respect to the decreased surface expression we detected upon aging, we additionally tested whether altered *Trem2* expression levels could be accountable for this observation (Supplementary material H). Notably, expression levels were low in the young CB but converged to those of the CTX in the middle-aged brain, conclusively suggesting the possibility of an increased shedding of TREM2 from microglial surfaces upon aging.

**Figure 2:**
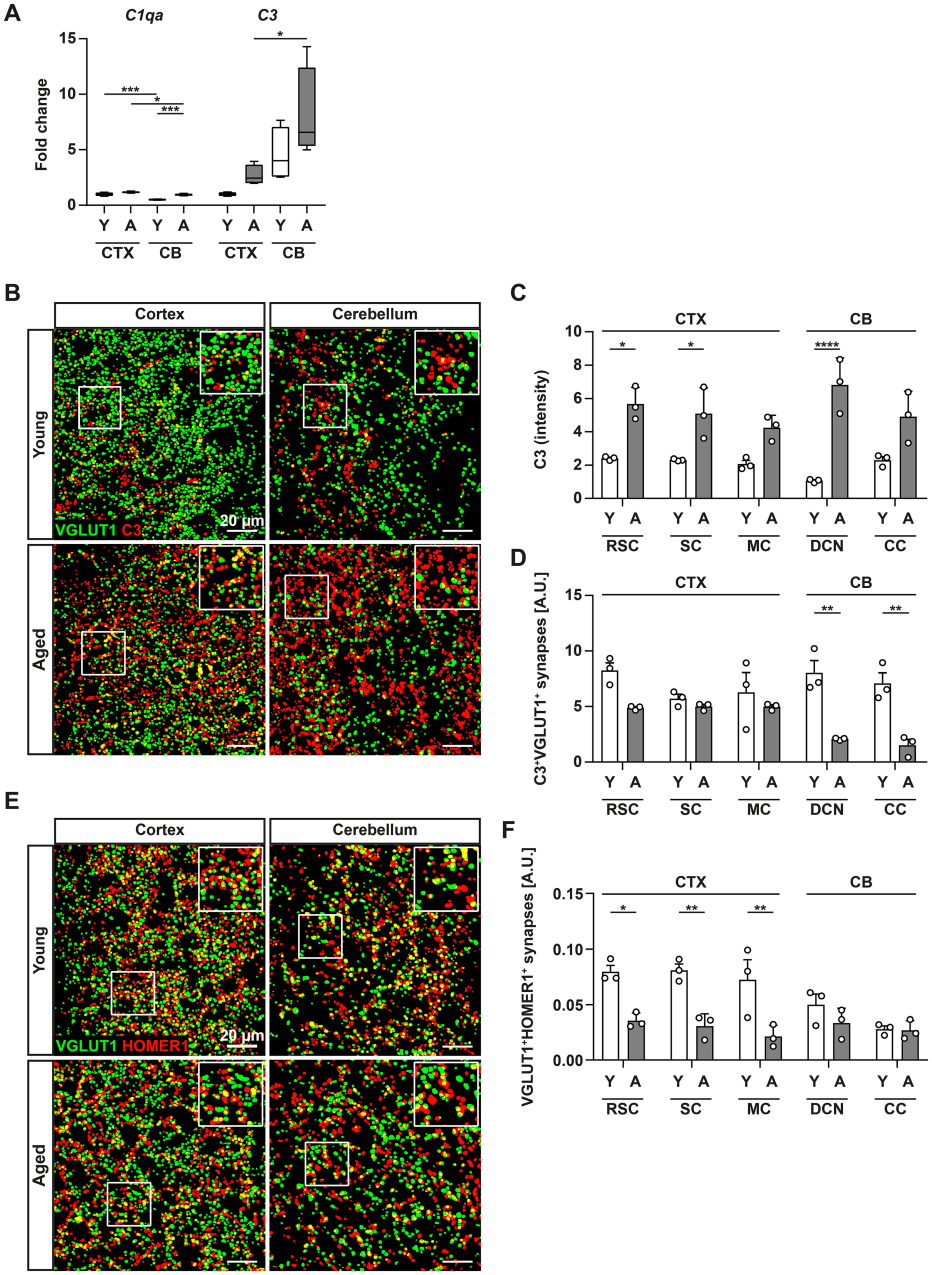
Increased expression of complement factors in middle-aged brains. **(A)** Gene expression of complement factors C1q (*C1qa*) and C3 (*C3*) was assessed by RT-qPCR in CTX and CB of young (white bars) and middle-aged (grey bars) mice. Expression level of target genes was normalized to the expression level of *Hprt* and subsequently, relative expression was normalized to the mean expression level in CTX of young animals to obtain the fold change. **(B)** Panels depict VGLUT1^+^ and C3^+^ puncta detected using the FIJI Synapse Counter plugin in CTX (left column) and CB (right column) after immunostaining with antibodies against VGLUT1 and C3 in young (upper panels) or middle-aged (lower panels) mice. **(C)** Bars represent quantified data for staining intensity of complement factor C3 in the different subregions of CTX and CB. **(D)** Bars illustrate the percentual overlap of C3^+^ and VGLUT1^+^ from data shown in **(B)** for the different subregions of CTX and CB. **(E)** Panels depict VGLUT1^+^ and HOMER1^+^ puncta detected using the FIJI Synapse Counter plugin in CTX (left column) and CB (right column) after immunostaining with antibodies against VGLUT1 and HOMER1 in young (upper panels) or middle-aged (lower panels) mice. **(F)** Bar charts illustrate quantified data of VGLUT1^+^HOMER1^+^ puncta from **(E)** for the different subregions of CTX and CB. Data are shown as mean ± SEM and were compared by two-way ANOVA with Tukey’s *post-hoc* correction. Significant differences are indicated by * *p* < 0.05, ** *p* < 0.01, *** *p* < 0.001, **** *p* < 0.0001.

**Figure 3:**
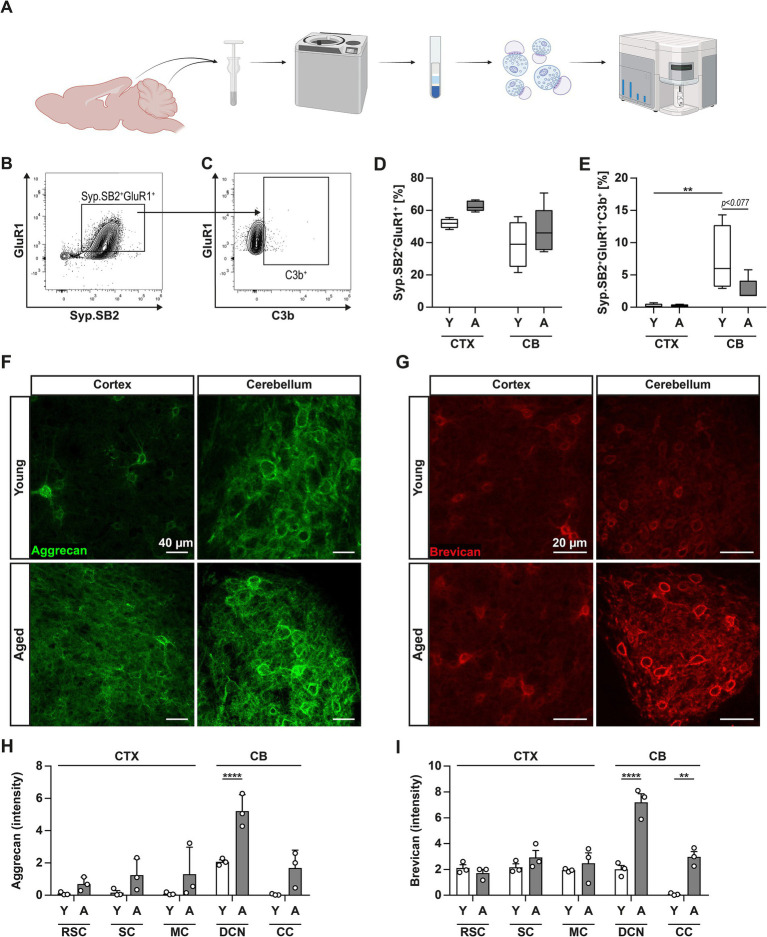
Elevated expression of ECM proteins in middle-aged CB. **(A)** Graphical illustration of synaptosome isolation from CTX and CB and subsequent analysis via flow synaptometry. **(B,C)** Representative gating strategy applied to synaptosomes from CTX to identify glutamatergic synaptosomes: Events were first gated on size (300–1,000 nm) and signal for FM4-64X (not shown), then for co-expression of presynaptic synaptophysin (Syp) and synaptobrevin 2 (SB2), and post-synaptic GluR1 (excitatory glutamatergic AMPA receptor). Finally, the population of Syp^+^SB2^+^GluR1^+^ synaptosomes was further gated on signal for complement factor C3b. **(D)** Whisker plots display the frequency of Syp^+^SB2^+^GluR1^+^ synaptosomes in CTX and CB of young (white bars) and middle-aged (grey bars) mice. **(E)** Whisker plots display the frequency of Syp^+^SB2^+^GluR1^+^C3b^+^ synaptosomes as percentage of the parent population (Syp^+^SB2^+^GluR1^+^, shown in **G**) in CTX and CB of young (white bars) and middle-aged (grey bars) mice. **(F,G)** Immunofluorescence imaging of CTX (left column) and CB (right column) stained against aggrecan and brevican in young (upper panels) or middle-aged (lower panels) mice. **(H,I)** Bar charts illustrate quantified staining intensity for aggrecan and brevican in the different subregions of CTX and CB. Data are shown as mean ± SEM and were compared by two-way ANOVA with Tukey’s *post-hoc* correction. Significant differences are indicated by * *p* < 0.05, ** *p* < 0.01, *** *p* < 0.001, **** *p* < 0.0001.

As resident macrophages and housekeepers of the brain, microglia perform typical phagocytosis-associated tasks such as the removal of debris and dead cells, but also exert an important role in the fine-tuning of neuronal circuits (Butovsky and Weiner, 2018). Through the engulfment and associated removal of synapses, microglia contribute to synaptic plasticity and normal function of the brain. This process is further regulated by factors of the complement system, which are required to tag synapses for the subsequent engulfment by microglia in multiple brain regions (Schafer et al., 2012; Stevens et al., 2007). In light of the upregulation of phagocytosis-related and lysosomal markers, especially in microglia from middle-aged CB samples, we next determined the gene expression levels of *C1qa* and *C3* (Figure 2A) in CTX and CB. In CBs of young animals, homeostatic expression of *C1qa* was found to be lower when compared to CTX. Despite displaying a marked increase in both regions upon aging, mRNA levels of *C1qa* remained significantly greater in CTX compared to CB. In contrast, the opposite was observed in the gene expression of *C3*, which was found highly upregulated in middle-aged CB but not CTX. Immunohistochemistry further supported these results, revealing a high prevalence of complement factor C3 both in middle-aged CTX and CB, as a basic prerequisite for synaptic pruning (Figures 2B,C; Supplementary material I). When closely examining the presence of C3-tagged glutamatergic synapses in both brain regions, we registered a significant decrease in VGLUT1^+^C3^+^ co-stained puncta only in the middle-aged CB (Figure 2D). This observation was paralleled by a reduction of VGLUT1^+^HOMER1^+^ synapses in CTX subregions upon aging whereas no difference was evident in the CB (Figures 2E,F; Supplementary material J). As this finding pointed towards a reduced rate of synaptic pruning in the CB, we were prompted to further evaluate the composition of complement-tagged excitatory synaptic terminals by performing flow synaptometry (Düsedau et al., 2021). To this end, we isolated crude synaptosomes from CTX and CB of young and middle-aged mice and immunolabeled complement factor C3b in addition to pre- (synaptophysin – Syp, synaptobrevin 2 – SB2) and post-synaptic (GluR1) components, respectively (Figures 3A–E). When we evaluated the overall frequency of Syp^+^SB2^+^GluR1^+^, our measurements could not confirm the previous results from IF as no significant differences among all experimental groups were detectable (Figure 3D), suggesting no prominent reduction of synapses in the excitatory circuitry to this point of aging. However, flow synaptometry revealed a substantially increased abundance of C3b^+^-tagged glutamatergic synaptosomes only in the CB of young mice with a trend to decline in middle-aged CB which was similar to our observation upon immunohistochemistry (Figure 3E).

In previous reports, we have shown the reciprocal interactions of glial cells and the ECM in the brain (Stoyanov et al., 2021; Cangalaya et al., 2024; Strackeljan et al., 2021). Hence, we wondered whether aging could affect the composition of the ECM in brain and modulate the microglia-mediated engulfment of synapses. Therefore, we immunostained brain sections against aggrecan and brevican, representing integral components of the brain ECM and being involved in the formation of peri-neuronal nets and the perisynaptic matrix (Frischknecht and Seidenbecher, 2012) (Figures 3F,G). When comparing the CTX subregions of young and middle-aged mice, we did not register any changes in the staining intensity of aggrecan and brevican (Figures 3H,I). In contrast, we noticed a considerable increase of both proteoglycans in the DCN and CC subregions of the CB upon aging. In summary, our experimental data demonstrated an aging-dependent increase of complement protein expression but did not detect a reduction of synapses, probably due to the simultaneous upregulation of ECM protein levels in the CB, mitigating synapse engulfment.

## 4 Discussion

Aging of the CNS is considered a driving factor of cellular senescence and is often accompanied by a decline of cognitive functions or the onset of neurodegenerative processes (Melo Dos Santos et al., 2024). On a cellular level, aging coincides with an increased release of pro-inflammatory signals that progressively render brain-resident microglia towards a higher activity state, possibly resulting in the re-activation of phagocytic pathways that lead to disruptive effects on synaptic neurotransmission. Brain regions have been reported to age at different velocities, with the murine CB to show signs of functional decline and loss of neurons at the age of 12 months and therefore earlier than other brain regions (McElroy et al., 2024; Morterá and Herculano-Houzel, 2012; Woodruff-Pak et al., 2010). However, only little is known about the mechanisms underlying these observed differences. In this study, we contribute to expanding existing knowledge by comparing the effects of aging between the cortex and the cerebellum. We detected region-specific differences in the cytokine milieu, complement factor expression and microglia activation state. Furthermore, we highlighted a striking reduction in complement factor C3-tagged glutamatergic synapses in the middle-aged CB, which was associated with the increased expression of ECM proteins potentially shielding synapses from microglia-mediated pruning during the process of aging.

It is understood that aging results in a progressively increasing low-grade inflammation of the brain (Franceschi et al., 2018; Finger et al., 2022). This is in line with our observation of increased IL-1β (CB) and TNF (CTX) gene expression levels in naturally aged animals. However, only in the CB this was paralleled by a reduction of TGF-β mRNA, suggesting impaired suppression of age-dependent inflammation in this brain region (Massagué and Sheppard, 2023). Given that the cytokines examined are attributed to the innate immune system (Lacy and Stow, 2011), we presumed that differences in their expression levels may be linked to changes in the activation state of microglia representing the primary population of brain immunocytes during homeostasis. Indeed, our results indicated a striking upregulation of phagocytosis-associated surface markers F4/80 and MHC I predominantly in the middle-aged CB. This finding further corroborated our initial findings and other studies demonstrating a greater shift in the phenotype and immune-amplifying genes of cerebellar microglia during aging (Hart et al., 2012; Grabert et al., 2016). In this context, scientific evidence proposes a causal link between the age-dependent priming of these cells and a decreased lysosomal proteolytic activity caused by lipofuscin depositions that occur with aging. This phenomenon results in increased oxidative stress and the induction of the p38 mitogen-activated protein kinase cascade (Nakanishi and Wu, 2009; Xu et al., 2008). Accordingly, we observed that microglia in middle-aged CB showed a prominent upregulation of the marker CD68, representing increased lysosome formations. Conversely, expression of surface markers CD45 and TREM2 was significantly reduced in both brain regions upon aging, whereas expression of the homeostatic marker CX3CR1 did not reveal any change. We, therefore, believe that, despite the overall increased activation, microglia in the CB do yet not correspond to their pathological counterpart of disease-associated microglia as seen in neurodegenerative conditions at this stage of aging (Keren-Shaul et al., 2017; Mrdjen et al., 2018; Deczkowska et al., 2018).

However, aging remains recognized as a major risk factor for the development of neurodegenerative diseases (Adamczyk-Sowa et al., 2022; Costantini et al., 2018; Jurcau et al., 2024; Ajoolabady et al., 2024) and large body of evidence emphasizes that the CB is not spared from pathologies like Alzheimer’s disease, Parkinson’s disease, and multiple sclerosis (Li et al., 2023; Samson and Claassen, 2017; Iskusnykh et al., 2024; McElroy et al., 2024). With reference to a previous report demonstrating the disproportional loss of synapses in 18- and 24-month-old mice and impairments of long-term depression developing between 4 and 8 month in the aging mouse CB (Woodruff-Pak et al., 2010), and our results suggesting a higher phagocytic capability of microglial cells in this brain region, we were prompted to further analyze the expression of complement factors *C1qa* and *C3* as a prerequisite for microglia-dependent synaptic pruning (Schafer et al., 2012; Vasek et al., 2016). Here, we detected significant increases of both, *C1qa* and *C3* levels, only in the CB upon aging, and a higher immunostaining of CB sections against C3 in aged mice. These observations are concordant with previous reports that have highlighted an aging-evoked upregulation of complement factor expression and their localization in close proximity to synapses (Bonasera et al., 2016; Stephan et al., 2013). It is now well established that components of the complement cascade play an important role in the microglia-dependent process of synaptic pruning during brain development via activation of the complement receptor 3 (Stevens et al., 2007; Schafer et al., 2012). In addition, complement signaling has been shown to mediate synapse and neuron elimination upon aging and disease (Stephan et al., 2012; Reichwald et al., 2009; Shi et al., 2015) but in the context of the aging CB, the active contribution of microglia to the elimination of synapses remains a subject for further investigation. This is attributable to the fact that the scope of our experiments did not extend to a detailed examination of *in situ* engulfment of synaptic terminals by these cells. Nonetheless, interactions of complement components with synaptic terminals or neurons are multifaceted and not necessarily reliant on direct involvement microglia. This notion is further supported by a recent study that highlighted the protective function of microglial soluble TREM2 (sTREM2) in suppressing the C1q-induced protease cascade that leads to deposition of C3 (Zhong et al., 2023). Here, the ectodomain of TREM2 is typically cleaved by the proteases ADAM10 and ADAM17, which results in the release of sTREM2 into the extracellular space. Intriguingly, we observed a reduced surface expression of TREM2 by microglia in both regions upon aging with an allover lower level in the CB when compared to the CTX. The obtained results could be suggestive of an augmented cleavage and shedding of TREM2 in the CB of aged rodents, particularly in consideration of the observation that *Trem2* gene expression in the CB and CTX of aged mice was found to be equivalent. Unfortunately, a limitation of our initial study design is the omission of the measurement of sTREM2 levels in brain tissue and we can only speculate whether induction of the classical complement cascade at the stage of C1q is suppressed or not.

Irrespective of this, high levels of C3 can facilitate its spontaneous hydrolysis into small C3a and large C3b fragments, which then either propel the complement signaling cascade or directly interact with their corresponding downstream complement receptors expressed on various cells of the CNS (Fatoba et al., 2022). In this context, a recent report has shown that astrocytes substantially increase NF-κB activity and concomitantly release C3 upon stimulation with pro-inflammatory cytokines, a scenario that is analogous to the inflammatory microenvironment we described in the aged CB. Secreted C3 then undergoes cleavage and the resulting C3a has been shown to impede the dendritic spine density through the action of the C3a receptor expressed on neurons (Lian et al., 2015). The importance of C3-mediated signaling cascades becomes even more pronounced in the context of neuroinflammation and –degeneration. Experiments in mouse models have shown that binding of C3a to its receptor induces STAT3 phosphorylation in microglia, thereby mediating memory dysfunction and elimination of excitatory synaptic terminals (Li et al., 2024; Litvinchuk et al., 2018). Notably, the expression of C3 and C3aR1 positively correlates with cognitive decline and further increases with progression of disease in the hippocampus of PS19 mice (Litvinchuk et al., 2018). In addition to its direct contribution to the tagging of synapses for phagocytosis or receptor activation, C3 also plays a pivotal role in the induction of a downstream signaling cascade that ultimately results in the formation of a membrane attack complex, which in turn leads to the creation of pores in cellular membranes. Interestingly, activation of these terminal complement pathways has been shown to directly modulate synapses under neurodegenerative conditions (Carpanini et al., 2022). Despite the limited amount of available information regarding complement factor and complement receptor expression in the aging CB, emerging evidence suggests that inhibition of C3ar1 signaling or STAT3 phosphorylation may offer therapeutic interventions to mitigate neuronal loss in the context of CB aging.

In consideration of the robust correlation between complement system elements and synapse loss, a comparative analysis was conducted on the quantity of complement-tagged synaptic terminals in aged mice. This analysis revealed a profound decline in C3^+^VGLUT1^+^ puncta within specific subregions of the CB. As an orthogonal approach to validate the observed changes, we performed flow synaptometry with synaptosomes isolated from CTX and CB (Düsedau et al., 2021). We harnessed the method’s ability to discern synaptic structures with intact pre- and post-synaptic terminals and found that C3b-tagged synaptosomes from the CB followed the same trend to decline as seen in immunofluorescence microscopy. Yet, no apparent change in the total population of glutamatergic synapses was detected in the aging CB, implicating that, despite the given prerequisites, synapses did not undergo microglia-mediated engulfment. To our knowledge, the mechanisms that could interfere with the attachment of complement factors to synapses are still poorly understood. With respect to the concept of synapses being embedded into a dense matrix of proteins and based on the results of our present and previous work, we speculated that the reduction in complement-tagged synaptosomes may be related to the ECM ameliorating the deposition of C3b.

In the past years, our reports have contributed to the understanding of the reciprocal interaction of glial cells and the ECM in the brain. We have shown the modulatory effects of ECM governing the microglial response to tissue damage (Stoyanov et al., 2021) and microglial-mediated synaptic remodeling (Cangalaya et al., 2024), and the capability of microglia cells to regulate the expression of ECM proteoglycans, thereby affecting the homeostasis of pre- and postsynaptic excitatory terminals (Strackeljan et al., 2021; Strackeljan et al., 2025). Still, the complete functionality of the ECM still remains unclear and ambiguous, especially in the context of aging. We therefore studied the levels of the proteoglycans brevican and aggrecan in the brains of aged mice and discovered a dramatically increased quantity of both in the CB. This is in line with the current understanding that aging of the brain is associated with cognitive impairment caused by a gradual decline in its plasticity due to an activity-dependent accumulation of ECM components (Fawcett et al., 2022; Dityatev et al., 2007). Brevican, which is primarily expressed in the perisynaptic zone, and other extracellular proteoglycans have been hypothesized to play a regulatory role in the propagation of the complement cascade (Frischknecht and Seidenbecher, 2012; Gialeli et al., 2018). In particular, brevican and aggrecan contain a C-terminal Sushi-like domain (or complement control protein module) which has been shown to moderate the complement pathway activation by targeting C3-cleaving enzymes (Gary et al., 2000; Tucker and Adams, 2024; Ojha et al., 2019; Gialeli et al., 2018). The reduced frequency of GluR1^+^C3b^+^ synaptosomes in the aged CB corroborates this hypothesis. Another feature of the ECM is represented by its capacity to regulate the diffusion properties of soluble factors (Dityatev et al., 2010; Frischknecht and Gundelfinger, 2012), and to bind chemokines and growth factors (Sutherland et al., 2023). This further raises the possibility that a denser network of proteoglycans in the aged CB may hinder the free diffusion of complement factors in the perisynaptic milieu (Tønnesen et al., 2023).

Yet, it should be noted that our flow synaptometry results exclusively address excitatory synapses, while a substantial population of inhibitory neurons, i.e., Purkinje cells, form the core of all cerebellar circuits. Accordingly, the protective effects of the ECM may not be equally applicable to all types of synapses and may not fully protect from the inevitable occurrence of synapse loss in the CB in later stages of aging. This poses the question of what alterations might occur over time in order to account for the shift from resilience to vulnerability. A potential correlation may be identified in the characteristics and activation pattern of microglia that are distinct in the CB as indicated by lower surface expression of CX3CR1. As previously demonstrated, the CX3CL1-CX3CR1 pathway is a pivotal mechanism that serves to downregulate the proinflammatory and neurotoxic capacity of microglia. In this regard, the CB has been shown to exhibit a lower neuronal expression of CX3CL1 (Sunnemark et al., 2005) while displaying a greater immune-alertness of microglia during aging (Grabert et al., 2016) and higher levels of cytokines under pathological conditions (Gaunt et al., 2023). However, further studies are needed to confirm whether this may result in a higher expression of ECM-degrading enzymes (Al-Roub et al., 2023; Rosenberg, 2002; Yong et al., 2001) and reduced protection of synapses by proteoglycans with the progression of CB aging.

In conclusion, our work highlights the regional, age-dependent differences in microglial and ECM properties in the CTX and CB. By linking our observations to the current state of knowledge, we propose a brain-specific mechanism that may be able to protect synapses from microglial engulfment. Therefore, future studies should focus on the underlying regulatory mechanisms that allow the ECM to modulate synaptic pruning in the brain, in order to develop novel therapeutic approaches to limit neurodegeneration.

## Data Availability

The original contributions presented in the study are included in the article/Supplementary material, further inquiries can be directed to the corresponding author.
