# Supplementary material for: Reduced synaptic tagging by complement protein C3 is associated with elevated extracellular matrix in the middle-aged cerebellum of mice
Source: Front Aging Neurosci. 2025 Jun 26;17:1616390. doi: 10.3389/fnagi.2025.1616390 (PMC12240999; doi:10.3389/fnagi.2025.1616390)
Supplement: Supplementary file 1 [file Table_1.docx]

##
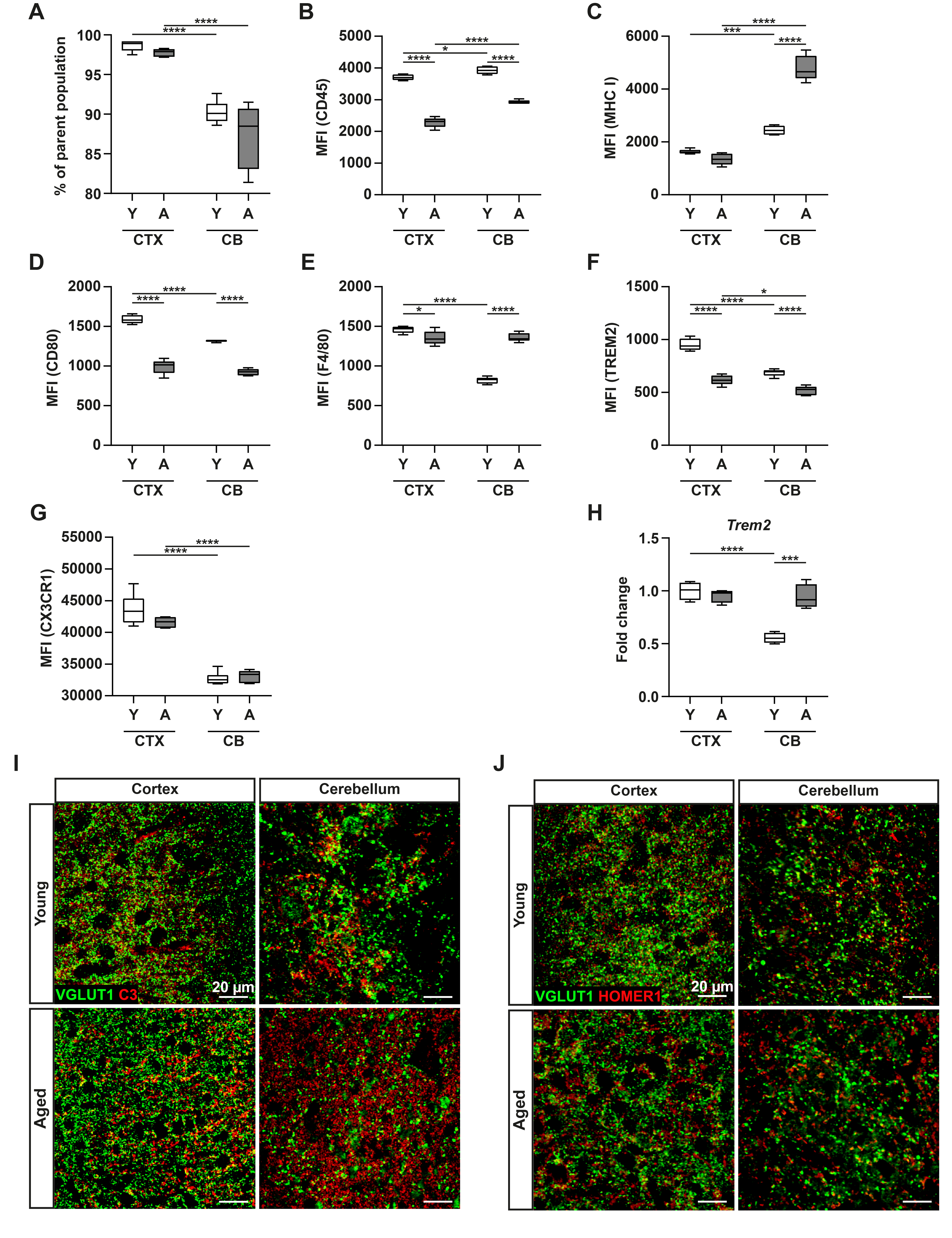


## Supplementary Material

(**A**) Flow cytometric characterization of microglia. Bar charts show the frequency of microglia in CTX and CB of young (white bars) and middle-aged mice (gray bars) as percentage of the parent population (single live cells) according to the gating strategy depicted in Fig. 1F-G. (**B-G**) Bar charts indicate the median fluorescence intensity (MFI) of microglial cells for the corresponding surface marker in CTX and CB of young (white bars) and middle-aged mice (gray bars). (**H**) Gene expression of TREM2 (*Trem2*) was assessed by RT-qPCR in CTX and CB of young (white bars) and middle-aged (grey bars) mice and thereafter normalized to the expression level of *Hprt*. Subsequently, relative expression was normalized to the mean expression level in CTX of young animals to obtain the fold change. (**I**) Representative immunofluorescence images of C3 and VGLUT1 staining. These images correspond to the input data for the C3 and VGLUT1 analysis shown in Fig. 2B, prior to processing with the FIJI Synapse Counter plugin. (**H**) Representative immunofluorescence images used for the synaptic puncta analysis. These images represent the input data for the quantification performed with the FIJI Synapse Counter plugin, as presented in Fig. 2E. Data are shown as mean ± SEM and were compared by two-way ANOVA with Tukey’s *post-hoc* correction. Significant differences are indicated by * (*p* < .05, ** *p* < .01, *** *p* < .001, **** *p* < .0001).
